# Calculation Methods of Solution Chemical Potential and Application in Emulsion Microencapsulation

**DOI:** 10.3390/molecules26102991

**Published:** 2021-05-18

**Authors:** Binkai Xu, Xiangdong Liu, Bo Zhou

**Affiliations:** 1 Jiangsu Key Laboratory of Micro and Nano Heat Fluid Flow Technology and Energy Application, School of Environmental Science and Engineering, Suzhou University of Science and Technology, Suzhou 215009, China; 15062305333@163.com; 2 College of Electrical, Energy and Power Engineering, Yangzhou University, Yangzhou 225127, China; liuxd@yzu.edu.cn

**Keywords:** emulsion microencapsulation, molecular simulation, chemical potential, Widom insertion, Metadynamics

## Abstract

Several new biased sampling methods were summarized for solution chemical potential calculation methods in the field of emulsion microencapsulation. The principles, features, and calculation efficiencies of various biased Widom insertion sampling methods were introduced, including volume detection bias, simulation ensemble bias, and particle insertion bias. The proper matches between various types of solution in emulsion and biased Widom methods were suggested, following detailed analyses on the biased insertion techniques. The volume detection bias methods effectively improved the accuracy of the data and the calculation efficiency by inserting detection particles and were suggested to be used for the calculation of solvent chemical potential for the homogeneous aqueous phase of the emulsion. The chemical potential of water, argon, and fluorobenzene (a typical solvent of the oil phase in double emulsion) was calculated by a new, optimized volume detection bias proposed by this work. The recently developed Well-Tempered(WT)-Metadynamics method skillfully constructed low-density regions for particle insertion and dynamically adjusted the system configuration according to the potential energy around the detection point, and hence, could be used for the oil-polymer mixtures of microencapsulation emulsion. For the macromolecule solutes in the oil or aqueous phase of the emulsion, the particle insertion bias could be applied to greatly increase the success rate of Widom insertions. Readers were expected to choose appropriate biased Widom methods to carry out their calculations on chemical potential, fugacity, and solubility of solutions based on the system molecular properties, inspired by this paper.

## 1. Introduction

**Figure 1:**
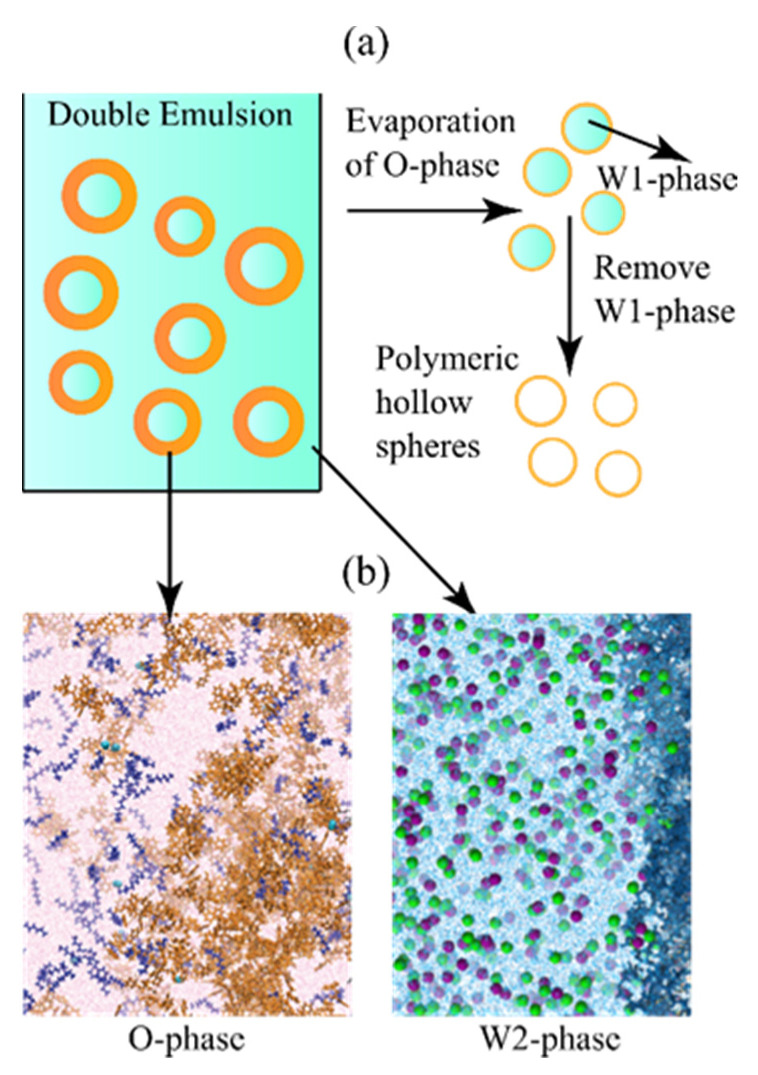
Illustration of (**a**) the emulsion curing process, and (**b**) the typical O-phase (polystyrene-octane in fluorobenzene), and W2-phase (NaCl-polyvinyl alcohol in water) in double emulsion for microencapsulation.

Emulsion microencapsulation is a technique to prepare hollow polymer microspheres with W1/O/W2 double emulsion. The emulsion is generated by dispersing the oil phase (O phase), which contains polymer macromolecules, and wraps the inner water phase (W1 phase) over the outer water phase (W2 phase). Hollow polymer microspheres can be obtained by curing the emulsion and removing the O phase solvent and the W1 phase. Hollow polymer microspheres are important polymer materials with an internal cavity, which can encapsulate various types of matter. An illustration of the W/O/W structure and the hollow sphere preparation process is shown in Figure 1a. In the fields of medicine and biology, Hollow polymer microspheres are widely used to encapsulate drugs to achieve long-distance macromolecule delivery and controlled release [1,2]. Due to the high refractive index difference between the spherical shell of hollow polymer microspheres and air, the spheres have a strong light-shielding ability and can be used in paint coatings in construction and leather, as well as in the cosmetics and paper industry [1,3,4]. In the energy field, hollow polymer microspheres can wrap phase change materials to prepare phase change microcapsules [5].

Our group carried out some research in the field of efficient preparation of double emulsions [6,7,8,9,10]. In the field of microfluidics, an effective microfluidic method based on a simple glass capillary assembly was proposed to encapsulate solids and prepare double emulsions, and visual experiments were performed to verify the effectiveness of the proposed microfluidic device. The results showed that the device controllably encapsulated solids in each double emulsion droplet produced, and the number of encapsulated solid cores could be well controlled by adjusting the flow rates of different phases. In order to improve the quality of the prepared microspheres, our research group studied the solidification and evaporation process of the double emulsion from a microscopic point. The dissolution and diffusion processes of water molecules on the W/O interface of the double emulsion were studied through molecular dynamics simulation. The dissolution and diffusion of droplets were closely related to the chemical potentials in the three dense and complex phases of a double emulsion. As shown in Figure 1b, typical emulsion for microencapsulation involved various molecules, where the O-phase contained polymer macromolecules as solidification material (i.e., polystyrene and poly-α-methyl-styrene), short-chain alkanes as regulating additives, and aromatics as solvent. The W2-phase acted as the curing environment, in which polyalcohol molecules were surfactant, and salt ions helped to inhibit the self-diffusion of water. The W1-phase was usually pure water or other filler that did not participate in the mass transfer process during emulsion curing.

Solution thermodynamics theory indicated that the chemical potentials, *μ*, of the three phases in a double emulsion were closely related to their Henry coefficients, vapor pressures, fugacities, free energies, and other parameters, which significantly affected the evaporation and curing processes of the double emulsion and the quality of the final shell products. The fugacity, *f*, of a fluid relates with the chemical potential in the form *μ = k_B_T* ln *f* + *C*, when *T* is the temperature, *k_B_* is the Boltzmann’s constant, and *C* is a constant related to the reference state of the fluid. The fugacity of a pure substance system is equal to the product of its pressure and fugacity coefficient, while the fugacity of a component in a mixture is equal to the product of the activity of the component and its pure substance fugacity. On the basis of Raoult’s law, in a sufficiently dilute solution, the activity coefficient of the solvent is unity at a constant temperature. Henry’s law explains that the fugacity of the solute is equal to the product of the Henry constant of the solution and the solute concentration when the solution is diluted. For emulsion systems containing multiple components, such as mixed nano-fluoride [11] and polymer emulsion [12], the fugacity, activity, and activity coefficients of each component of the infinitely dilute solute can be measured by gas chromatography. Kim et al. [13] measured the activity coefficient of solvent under the infinite dilution of the monodisperse polystyrene-hydrocarbon system in the temperature range of 373.15–423.15K by gas chromatography. Zlem et al. [14] carried out the retention volume diagrams of ethyl acetate, isobutyl acetate, tert-butyl acetate, benzene, n-hexane, n-heptane, n-octane, acetone, chloroform, and acetonitrile on poly hexyl methacrylate (PHMA) by reversed-phase chromatography and computed the weight fraction activity system of polymer-solvent when PHMA was diluted infinitely. The activity coefficients in binary or ternary polymer solutions under infinite solute dilution could be readily measured. However, during the process of evaporation, the concentration distribution of polymer emulsion changed over time, so it was necessary to measure the fugacity of each component at various finite concentrations, except for the infinitely dilute solutions. Computer simulations were proper solutions for these hard and expensive tasks, while the chemical potential of emulsion systems containing polymers and various additives could be calculated. Therefore, to correctly understand the influence of various molecules on the chemical potential of the emulsion and even propose optimized additive compositions, it was necessary to develop accurate and efficient solution chemical potential calculation methods.

The Widom insertion method [15], as the original method for chemical potential calculation in the field of computational chemistry, has been verified by a large number of studies as the main method to calculate the excess chemical potential of molecular systems. For example, Vrabec et al. calculated the chemical potential information of quantitative Lennard–Jones fluids [16]. Schnabel et al. calculated the residual chemical potential of infinitely diluted solutes (methane, nitrogen, oxygen, and carbon dioxide) in ethanol solvents in the range of 273–498 K by the Widom insertion method and calculated Henry’s law constants [17]. Wu et al. calculated the Henry constants of nitrogen, oxygen, methanol, and carbon dioxide in ethylene oxide and ethanol liquids [18]. Gestoso et al. studied the chemical potential and solubility of nitrogen, oxygen, argon, and carbon dioxide in 1-magnetic-4-polybutadiene [19]. The conventional Widom method could successfully solve the chemical potential calculation in low-density systems but got in trouble for dense and complex solution systems with short intermolecular distances because of the low success rate of the original insertion method [20]. Thus, the conventional Widom insertion method could hardly deal with the chemical potential calculation required by the analysis, and optimization of the complex solutions used in the emulsion microencapsulation technique. Therefore, the researchers optimized the Widom method continuously by introducing various biases for Widom insertions. Lee et al. divided the space into a strong adsorption region and a weak adsorption region and inserted particles in the strong adsorption region to accurately calculate the Henry coefficient [21]. Perego et al. calculated the chemical potential of density liquids and non-homogeneous liquids using the Metadynamics method [22,23]. Li et al. calculated the solubility of sparingly soluble solutes and sparingly soluble organic/inorganic materials using the extended Einstein crystal method [24,25]. Rosenbluth et al. provided a method for long-chain molecule insertion, which could improve the success rate of Widom’s insertion method [26]. All the optimization methods mentioned here, as the bias sampling methods of Widom insertion, were divided into three categories: (1) volume detection bias, (2) simulated ensemble bias, (3) particle insertion bias.

From the aspects of optimizing the system configuration and improving the sampling success rate, this paper analyzed and summarized the latest progress of chemical potential calculation technique originated from the Widom insertion method. Additionally, an optimized volume detection bias method was proposed on the basis of a literature review, and the verification calculation was carried out for liquid water, argon, and fluorobenzene systems. Several suggestions on the selection of biased Widom methods were proposed for different solution systems in the O-phase and W2-phase of microencapsulation emulsion, based on the practice in the literature and our analysis of the algorithm features. The paper was organized as follows: the original Widom method was introduced and analyzed in Section 2, three types of biased Widom method were introduced and investigated in Section 3, and the conclusions were given in Section 4.

## 2. Widom Insertion Method

### 2.1. Principle

**Figure 2:**
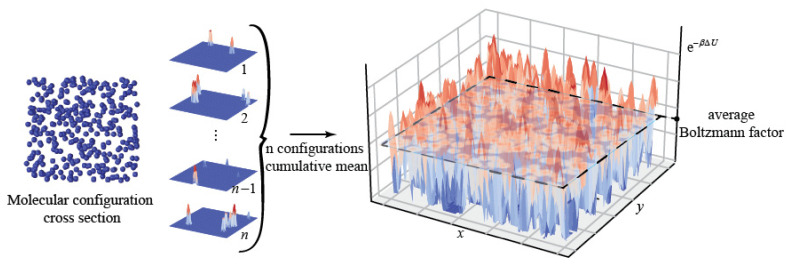
Calculation of the average Boltzmann factor with conventional Widom method.

The Widom insertion method is a classical method for chemical potential calculation of pure substances or solutions [15]. According to the summary given by Frenkel [20], the chemical potential of a particle (atom, molecule, ion, etc.) system can be decomposed as follows:

(1) $$\mu =\mu_{\mathrm{id}}(\rho )+\mu_{\mathrm{ex}}$$

(2) $$\mu_{\mathrm{ex}}=-k_{B}T\ln\int \langle \exp(-\beta \Delta U)\rangle_{N}{\mathrm{ds}}_{N+1}$$

where *μ*_id_ (*ρ*) is the chemical potential of an ideal gas with a density *ρ*, which can be calculated by thermodynamics. The core manipulation of this method is to insert a test particle into a system of *N* particles, which increases the particle number of the system from *N* to *N* + 1 and changes the internal energy of the system by Δ*U*. The denotation 〈…〉*_N_* refers to the average of a physical quantity in an *N*-particle canonical ensemble. When calculating *μ*_ex_ with Equation (2), test particle insertions were attempted over all positions of the internal space of the system, and the ensemble average, 〈e^(−*β*Δ*U*)^〉*_N_*, was integrated. As shown in Figure 2, test particles were randomly inserted on the cross-sections of multiple molecular configurations in a canonical ensemble, and the Boltzmann factor, e^(−*β*Δ*U*)^, at the insertion positions was calculated, and then averaged for all configurations, which led to the integration of 〈e^(−*β*Δ*U*)^〉*_N_* in Equation (2).

The *μ*_ex_ calculation process based on Widom insertion involves two key sampling processes: one is to extract molecular configurations from the ensemble, and the other is to decide the insertion position of the test particles. For the conventional Widom method, the insertion positions of the test particles are randomly generated following a uniform distribution. A sampled insertion position with a Boltzmann factor close to 0 is considered to be a failure since the associated Boltzmann factor almost contributes nothing to the integration and averages in Equation (2). On the contrary, a sampled position whose Boltzmann factor is significantly greater than 0 is considered to be a successful insertion. A specified configuration contains several appropriate positions for successful insertions, so the integration of the Boltzmann factor would be underestimated if any of the appropriate positions were missed by the sampling algorithm. As a result, much more insertions must be attempted for systems with high densities to avoid missing appropriate insertion positions and non-zero Boltzmann factors. Therefore, improving the calculation accuracy and efficiency of the average Boltzmann factor requires: (1) a higher success rate of insertion in a single configuration and (2) a lesser number of configurations to be analyzed in the ensemble. An important means to optimize the Widom algorithm is to adjust the sampling rules for the insertion positions and reduce occurrences of failed sampling of the positions that hardly contribute to the Boltzmann factor, that is, to apply biased sampling of the insertion positions of test particles. Concretely, there exists a variety of biased sampling ways, such as pre-judging the insertion position to exclude overlapping positions or constructing molecular configurations that are conducive to insertion. The bias sampling modes for the Widom method are divided into three categories here: (1) volume detection bias, (2) simulated ensemble bias, (3) particle insertion bias, which was introduced in Section 3.

### 2.2. Application of the Conventional Widom Method

The Widom method has been widely used in the calculation of solution chemical potential, whose original algorithm was easy to be realized by a computer program. Several applications of the conventional Widom method in solution systems were reviewed here, while the simulation features are listed in Table 1. Wu et al. calculated the Henry constants of nitrogen, oxygen, methanol, and carbon dioxide in ethylene oxide and ethanol liquid with the canonical ensemble (NVT) configurations generated by Monte Carlo simulation (MC) and molecular dynamics (MD), based on an AA whole atomic force field or a UA combined atomic force field, and the results deviated from the experimental data when the solvent density was too high [18]. Xuan et al. calculated the chemical potential and solubility of carbon monoxide in ethanol with the Widom method [27]. Gestoso et al. investigated the chemical potential and solubility of nitrogen, oxygen, argon, and carbon dioxide in 1-magne-4-polybutadiene with 30 or 300 chain lengths and the relative deviations between simulation data, and the experimental results were less than 3% [19]. The solubility of nitrogen, oxygen, and argon in long-chain solvents was more consistent with the experiment data, while the solubility of carbon dioxide in short-chain solvents was more consistent with the experimental data, so the authors speculated that the chain length of polymer affected the accuracy of the simulation results by affecting the success rate of the small molecule insertion. Albo et al. calculated the chemical potential and solubility of naphthalene in supercritical carbon dioxide, which gave non-consistent results with the experimental data due to inappropriate force field parameters [28]. Coskuner and Deiters calculated the excess chemical potential of tritium molecules in water [29], Carrero-Mantilla et al. computed the Henry constant of hydrogen in cyclohexane and benzene [30], and Pai et al. calculated the excess chemical potential of naphthalene, benzoic acid, and phenanthrene in carbon dioxide through canonical ensemble Monte Carlo simulations [31].

These results showed that the conventional Widom algorithm could effectively calculate the chemical potential and many other extended parameters of simple molecules in relatively sparse systems if appropriate force fields were employed. A significant fact is that although both the simulation area and the number of molecules involved in the above studies were small, the number of insertion position sampling required by the calculation was very large, as shown in Table 1. Since the distances between insertion positions of the Widom test particles and the existing atoms were always very close in a dense system, the internal energy of the system changes greatly after the test particles were inserted, and most of the Boltzmann factors were almost zero. More configurations and more insertion attempts must be involved to achieve good results. The biased sampling method was expected to accelerate the conventional Widom method and enhance the practicability of this computer simulation technique.

## 3. Optimization of Widom Method Based on Biased Sampling

### 3.1. Volume Detection Bias

**Figure 3:**
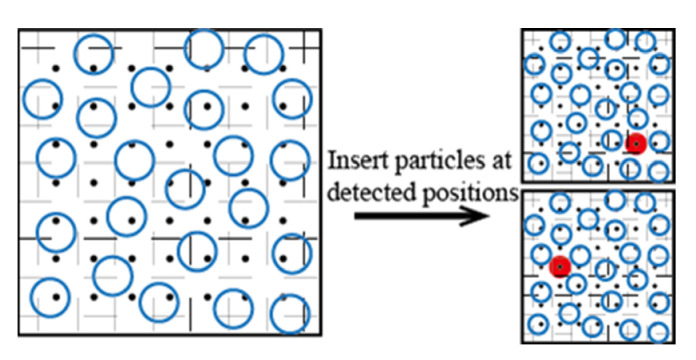
Illustration of the volume detection bias method.

The volume detection bias [34,35] is to insert a test particle B (represented as a black point, denoted as B-particle) in a grid uniformly meshed in the configuration to evaluate whether the present grid volume is suitable to insert a test particle A (represented as a red solid circle, denoted as A-particle) to calculate the chemical potential of the A-particle, as shown in Figure 3. The A-particle is usually an actual atom or molecule, but the B-particle can be a virtual particle having an artificial potential. Various physical quantities for the detection of grids can be selected according to the particle and force field type of the system. For example, Lee et al. divided the space into a strong adsorption region and a weak adsorption region according to the binding energy of X/MOF and inserted the A-particle with a bias in the strong adsorption region to calculate the Henry coefficient of X (X= carbon dioxide or nitrogen or methane or ethylene) in M-MOF-74 (M= Zn or Mg) [21]. Khawaja et al. calculated the solubility of a gas in nitrile butadiene rubber using the local molecule number density as the physical quantity for detection [36]. Yang and Whiting calculated the solubility of methane and n-butane in polypropylene, propyl-methyl-siloxane, and 4-methyl-2-pentyne on the basis of the local particle number density [37]. Zanuy et al. calculated the solubility of helium, argon, and methane in a poly-α-alkyl-β-L-aspartate solution based on the intermolecular distance [38]. Comparing with the unbiased Widom insertion technique, the volume detection bias could provide more than 40 times the computational efficiency in some situations, and the deviation between the simulated value and the measured value was less than 5%, as shown in Table 2.

Taking the local particle number density as a criterion, this kind of bias method mainly includes the following steps:

1. Mesh the system with a volume of V into N grids uniformly; 

2. Insert B-particles into each grid and calculate the distances between the B-particles and the existing particles in the system, and the local particle number density of the grid point is calculated according to the above distances; 

3. Judge whether it is suitable to insert the A-particle into the grid according to the local number density of each grid. The total number, *N*_fv_, and the total volume, *V*_fv_, of the grids, which are suitable for insertion, are determined. The excess chemical potential of A-particle, *μ*_ex_revised_, can be revised and written as:

(3) $$\mu_{\mathrm{ex}\_\mathrm{revised}}=-\frac{N_{\mathrm{fv}}}{N}k_{B}Te^{-\beta \mu_{\mathrm{ex}}}$$

where *μ*_ex_ is the excess chemical potential calculated in the grids, which are detected and evaluated as appropriate insertion grids. Multiple insertions can be made in each appropriate grid to further improve the calculation accuracy; when the A-particle is randomly inserted into the appropriate grids, the revised excess chemical potential can be written as:

(4) $$\mu_{\mathrm{ex}\_\mathrm{revised}}=-\frac{V_{\mathrm{fv}}}{V}k_{B}Te^{-\beta \mu_{\mathrm{ex}}}$$

**Figure 4:**
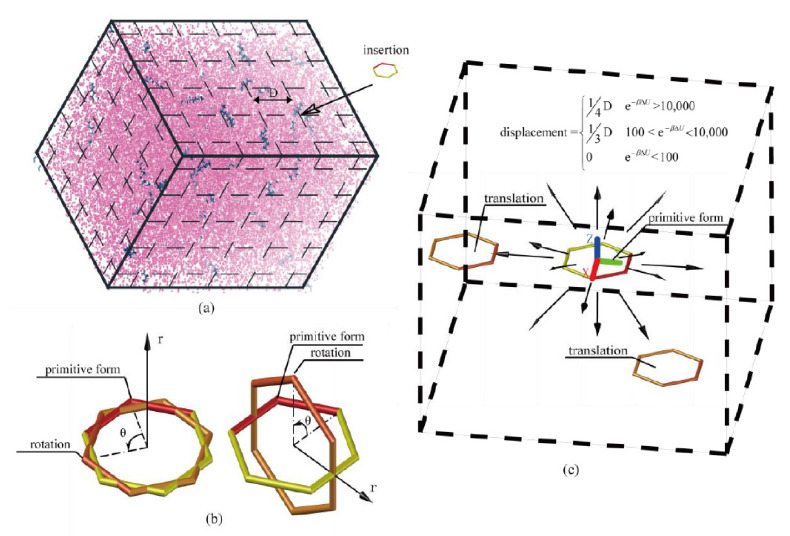
Operation of the inserted probe molecule for detailed average inside a grid. (**a**) Inserting a probe molecule. (**b**) Rotating the inserted probe molecule. (**c**) Translating the inserted probe molecule with a displacement that depends on the local Boltzmann factor.

Here, we proposed a further optimized volume detection bias method based on a two-level criterion and detailed average. The chemical potentials of pure water and argon were calculated with this detailed average Widom method for validation. In this procedure, when a B-particle was inserted into a grid center, we first checked whether it overlapped with other atoms in the system, which was the first level criterion. We further assessed the insertion position passing the B-particle overlap check by evaluating the Boltzmann factor of an independent A-particle inserted at the same position, while this insertion position was regarded to pass the second level criterion if the Boltzmann factor exceeded some certain values. As shown in Figure 4c, the A-particle satisfying the two-level check was translated slightly for 14 times inside the grid along the following directions: (1,0,0), (−1,0,0), (1,0,0), (0,1,0), (0,−1,0), (0,0,1), (0,0,−1), (1,1,1), (1,1,−1), (1,−1,1), (1,−1,−1), (−1,1,1), (−1,1,−1), (−1,−1,1), (−1,−1,−1), while the translation distance depended on its Boltzmann factor and the grid size, *D*. If the A-particle was a polyatomic molecule, it was further rotated in 17 forms at each translated position. The rotation patterns for a molecule included: 1) rotated 90, 180, or 270 degrees with the rotation axis (1,0,0), (0,1,0), or (0,0,1); 2) the particle is rotated 120 or 240 degrees with the rotation axis (1,1,1), (1,1,−1), (1,−1,1), or (1,−1,−1). With these efforts, the insertion grid was detected in great detail since proper insertion patterns of a probe molecule were searched as much as possible. All the Boltzmann factors for the 14 × 17 insertion patterns in a detected grid were averaged. Comparing to other volume detection bias methods mentioned previously, the present procedure provided a more accurate Boltzmann factor for the insertion position with the same computing power, especially for line or plane molecules in dense systems because the insertion pattern of the molecule was carefully processed.

Several liquid systems were investigated by the two-level volume detection bias method proposed in this work. To verify the method, two systems consisting of 3375 water molecules (TIP3P) and 512 argon atoms were constructed. 50 equilibrium and independent configurations generated by MD were selected for Widom insertion, and each configuration was meshed as 50 × 50 × 50 grids with a grid volume of 1 Å^3^. TIP3P water and argon atom were used as both A-particle and B-particle for the two systems. The first level criterion was that the minimum distance between the inserted probe molecule and any other atom was greater than 1 Å. The second level rule was that the grid was only investigated further if the Boltzmann factor of the probe molecule was greater than 100, while the translation distance for detailed average was 1/3 *D* or 1/4 *D* in the cases of 100 < *e^−β^*^Δ*U*^ < 10,000 or *e^−β^*^Δ*U*^ > 10,000. The calculation results for the excess chemical potentials were shown in Table 3, where *μ*_ex___R_ was calculated by formula *μ*_ex_ = *k*_B_*T*ln(*zϕ*), while the fugacity coefficient, *ϕ*, and the compression factor, *z*, was provided by National Institute of Standards and Technology (NIST) data [40]. *μ*_ex_W_ is the original data calculated by the optimized volume detection bias method. *μ*_ex_p_ and *μ*_ex_c_ are the long-range corrections to pairwise van der Waals forces and Coulombic forces [20]. The corrected *μ*_ex_ is the sum of *μ*_ex_W_, *μ*_ex_p_, and *μ*_ex_c_. Comparing *μ*_ex_ to *μ*_ex___R_, the difference between the calculation results of the improved Widom method and NIST data was less than 4% for all liquids, which showed good accuracy of the present algorithm. The computation efficiency could be further improved by optimizing the first level criterion, such as using a more efficient algorithm to check the atom overlapping or design suitable potentials for B-particles.

**Figure 5:**
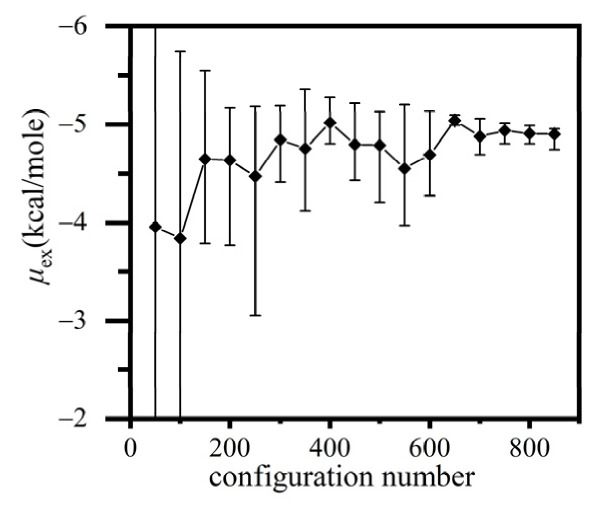
The excess chemical potential of liquid fluorobenzene at 1 atm and 320 K converges with the increase of configurations.

The excess chemical potential of fluorobenzene, as a common solvent in emulsion microencapsulation processes, was calculated with the verified two-level detailed average Widom method. The drawback to applying the conventional Widom method for this type of molecules was that the calculation usually underestimated *e^−β^*^Δ*U*^ because the proper insertion patterns in a grid were rare and, hence, could be very likely missed when the probe molecule was randomly rotated at the grid center. In the present detailed average method, the insertion pattern of A-particles was fully scanned inside the grid by performing uniform translations and rotations. The fluorobenzene liquid system consisted of 12,000 atoms, and all the configurations for Widom insertion were meshed as 50 × 50 × 50 grids. Figure 5 shows the convergence of *μ*_ex_ as more configurations were calculated. The calculation required much more configurations to achieve a satisfying statistical accuracy, comparing to water or argon, since the grids that met both of the two-level criterions were much less than those in simpler systems. The uncertainty of *μ*_ex_ was calculated as the standard deviation of the sequences consisted of *μ*_ex_ averaged in an ensemble of every 50 configurations. The simulated *μ*_ex_ converged to −4.93 ± 0.11 kcal/mol, which was close to the calculated result, −4.23 kcal/mol, predicted by Equation (1) where the chemical potential, *μ*, was estimated by approximating the fugacity with the saturation vapor pressure, 26.40 kPa [41].

In summary, the volume detection bias methods had higher accuracy than the conventional Widom method, which significantly reduced the number of insertion attempts and effectively improved the calculation efficiency. However, it took a certain amount of computation to insert the probe particle and evaluate the insertion position before inserting the particle formally, so it was necessary to design a reasonable procedure to evaluate the detection space. These methods were suitable for homogeneous systems with medium and high density, such as dilute aqueous solutions as the W1 and W2 water phases in a double emulsion system.

### 3.2. Simulation Ensemble Bias

Simulation ensemble bias is to adjust the evolution rules of a system to generate appropriate configurations for Widom insertion. The grand canonical Monte Carlo (GCMC) method is a common method of simulation ensemble bias, which evolves a system governed by its chemical potential [20]. In the GCMC method, the temperature and volume of the system remain constant, and the number of particles is dynamically changed to push the chemical potential to the set value, involving a large number of particle insertions and deletions during the evolution process. Although the GCMC method is widely used, it cannot calculate the chemical potential of a specified system configuration directly. Alternatively, achieving the desired configuration by adjusting the chemical potential gradually is a practicable way to fix the chemical potential, but the computation cost may be very expensive.

Another approach of simulation ensemble bias is to generate configurations containing predesigned and appropriate particle insertion positions. Such configurations may not be physically valid. Simulation systems can generate uniformly distributed low-density areas and randomly distributed high-density areas at the same time through the external potential field designed by Powles and Moore. Since the chemical potential in an equilibrium system is homogeneous, the chemical potential in the low-density areas is equivalent to that in the high-density areas [42]. Moore et al. further proposed to apply an external potential field that matches the periodic boundary conditions of the simulation to enhance the system density variation in a periodic manner in the whole system [43].

The latest progress of this method is the Well-Tempered(WT)-Metadynamics method [22]. Comparing to the approach of Powles and Moore, the WT-Metadynamics method can synchronously compute the chemical potential during the system evolution. This method constructs a family of scalar functions {$s_{n}^{r}$(*R*)} with an offset external potential sequence {*V_n_*(*s*)}, and iterates them recursively in the dynamic evolution [22,44]:

(5) $$s_{n}^{r}(R)=-\beta^{-1}\ln(\frac{1}{M}\sum_{i=1}^{M}\exp[-\beta \Delta U_{n}^{r}(R_{i}^{*};R)])$$

(6) $$U_{n}^{r}(R)=U(R)+V_{n}^{}\left[s(R)\right]$$

(7) $$V_{n}^{}(s)=V_{n-1}^{}(s)+G(s,s_{n})\exp[-\frac{1}{\lambda -1}\beta V_{n-1}(s_{n})]$$

The arithmetic average chemical potential of the test particles inserted at *M* exclusion cores is denoted as $s_{n}^{r}$(*R*). The subscript *n* is the number of iterations, and the superscript *r* indicates that the bias potential energy *V_n_*(*s*) is switched on in the calculation of the system energy. *R* is the set of coordinate vectors of all particles in the system. *M* test particles are uniformly and periodically distributed in the system, and their coordinate set is R*. Recursive iteration of the bias potential energy involves a relaxation factor of a Gaussian function: *G*(*s*,*s*’) = *ω*exp [−(*s* − *s*’)^2^/2*σ*^2^]. *V_n_*(*s*) and $s_{n}^{r}$(*R*) are iterated within a certain time interval of the whole dynamic evolution. With strict proof [45], the function sequence *V_n_*(*s*) eventually converges to:

(8) $$V(s)=-(1-\frac{1}{\lambda })(-\beta^{-1}\ln P^{r}(s)+C)$$

where *C* is a constant, *P^r^*(*s*) is the probability density of a scalar function *s* in the ensemble with the bias potential *V*(*s*). Additionally, *P^r^*(*s*) has the following relationship with *P*(*s*), the probability density of *s* in the ensemble without bias potential energy:

(9) $$P^{r}(s)=\frac{{\left[P(s)\right]}^{1/\lambda }}{\int ds^{\prime }{\left[P(s^{\prime })\right]}^{1/\lambda }}$$

Using the above method, the probability density *P^r^*(*s*) in the bias potential ensemble could be calculated. Then, *P^r^*(*s*) was corrected by the weight factor *λ* to obtain the probability density *P*(*s*) without bias potential energy, and the chemical potential of the system without bias potential energy could be calculated.

**Figure 6:**
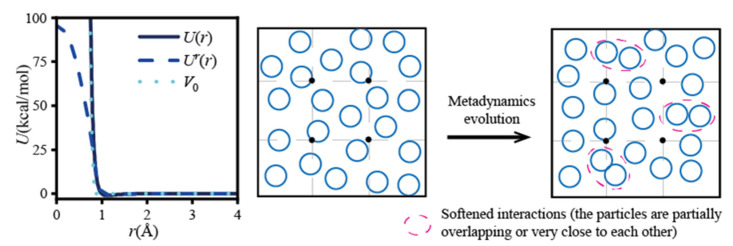
The external potentials and system evolution of the WT-Metadynamics method.

**Figure 7:**
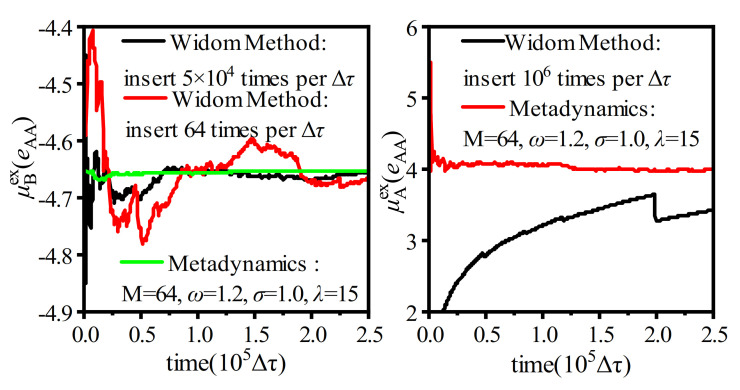
Chemical potential calculation with WT-Metadynamics in a high-density binary Lennard-Jones system (redrew with data in [22]).

The key operation of the chemical potential calculation with the WT-Metadynamics was to set the appropriate bias potential energy *V_n_*(*s*) to soften the pairwise interaction so that the particles could be close to and even overlap with each other to a certain extent (Figure 6). This approach subtly reduced the density of the system around the R* positions, increased the probability of cavitation generation, and decreased the potential energy increment of the pre-positioned particles at the R* positions, which was equivalent to achieve several successful Widom insertions. For example, the bias potential energy *V*_0_ as shown in Figure 6 (left) was used to bias the particle interaction *U*(*r*) to *U^r^*(*r*) for a high-density binary Lennard–Jones (LJ) system, where *r*_0_ was an artificially specified characteristic distance between particles and the value was 0.88 times of the LJ characteristic length. The simulation system contained 640 A and 160 B particles, the simulation time was 2.5 × 10^5^Δ*τ*, and the result is shown in Figure 7 [22].

As shown in Figure 7, the convergence rate of the chemical potential calculation with the WT-Metadynamics method was much faster than that of the Widom method with two types of insertion attempts within the same simulation time. Moreover, the convergent result could be obtained by using the WT-Metadynamics method, while the conventional Widom method failed. In addition, the WT-Metadynamics method was proven to be applicable to non-uniform systems [23]. Based on the reported practices, the WT-Metadynamics method showed stronger applicability to high-density systems with complex local structures. However, the implementation of this algorithm required a reasonable selection of the bias potential function *V_n_*(*s*) and relevant Gaussian function parameters. Osmair calculated the discrete free energy of the sugar chain of cellulase Cel48F-sugar in the catalytic channel by the metadynamics technique. The width of the Gaussian was 0.05 nm, and the height of Gaussian was 0.5 kJ/mol. The discrete free energies of WT-Cel48F, Q543A-Cel48F, and E542A-Cel48F were 62.1 kcal/mol, 45.9 kcal/mol, and 41.8 kcal/mol, respectively, and the errors of all free energies were 1–2 kcal/mol [46]. Bjelobrk et al. calculated the solubility of urea and naphthalene in methane cyanide, ethanol, and methanol solutions by metadynamics. The simulation and experimental results clearly showed that, in all cases, the simulation could correctly predict the order of magnitude of the solubility value. In addition, the solubility trends of urea and naphthalene in different solvents were also correctly predicted [47]. Biswas and Wonguse simulated the deprotonation kinetics of acetic acid in the bulk phase and air-water interface environment. The distance between the O-atom and H-atom of the acetic acid hydroxyl group was taken as the set variable, and the Gaussian width and height were set to 0.1 Hartree and 0.0005 Hartree, respectively. The simulated results of the deprotonation free energy of the acetic acid at the air-water interface were consistent with the existing experimental data [48]. Salvalaglio et al. calculated the free energy change of the phase transition of urea in an aqueous solution so as to observe the nucleation of urea in an aqueous solution [49]. This method had a great potential in calculating the chemical potential of the oil phase solution containing various polymer solutes in double emulsion systems.

### 3.3. Particle Insertion Bias

In the emulsion microencapsulation field, each phase may contain various passivators and surfactants, which regulate the evaporation process of double emulsions such as ions, short-chain alkanes, and poly-alcohols. The sizes of these particles were significantly larger than that of the solvent molecules, which further reduced the success rate of the Widom insertion when investigating the chemical potentials of these components.

The idea of particle insertion bias was to improve the success rate of every single insertion to reduce the total number of insertion attempts. This kind of bias was mainly suitable for high-density systems with macromolecular solutes; here, the extended Einstein crystal method [24,25] and Rosenbluth sampling [26] were introduced.

#### 3.3.1. Extended Einstein Crystal Method (EECM)

**Figure 8:**
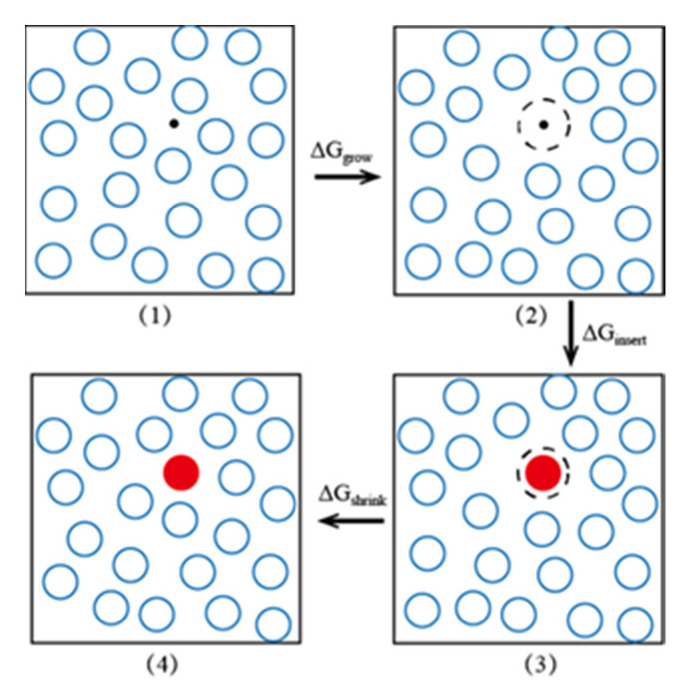
EECM method diagram. The blue hollow circles are solvent molecules, the red solid particles represent solute molecules, and the black dotted lines are cavities induced by the repulsive particle.

EECM method is a new sampling method proposed by Li and Frenkel in 2017 [24,25], which includes the following steps:

1. A particle that repels the solvent molecules is inserted into the solution to generate a cavity, and the free energy change caused by this operation is denoted as Δ*G*_grow_, as shown in Figure 8 (1) and (2).

2. The repulsive particle is removed, and a solute molecule is inserted at the same position, which causes another free energy change, Δ*G*_insert_, as shown in Figure 8 (2) and (3).

3. After relaxation, the solution shrinks; the free energy changes by Δ*G*_shrink_, as shown in Figure 8 (3) and (4).

Finally, the solution excess chemical potential can be expressed as:

(10) $$\mu_{\mathrm{ex}}=\Delta G_{\mathrm{grow}}+\Delta G_{\mathrm{insert}}+\Delta G_{\mathrm{shrink}}$$

Based on a simple extension of the EECM method and an all-atomic force field, the solubility of naphthalene in water was calculated by Li et al. using LAMMPS software [25,50]. The solubility of acetaminophen in ethanol solution was calculated by Bellucci et al., showing good agreement with the experimental solubility [51]. In addition, EECM was derived and proven by Gobbo et al. that it could be used to calculate the solubility of macromolecules in insoluble systems and biomedical force fields [52]. Noya et al. calculated the free energy of ice XIII and ice XIV by the Einstein crystal method. The calculated results of free energy were consistent with those of the thermodynamic integral of the equation of state [53]. Caballero et al. calculated the complete phase diagram of symmetrical colloidal electrolytes by the Einstein crystal method and Monte Carlo simulation [54]. Blas et al. calculated the free energy of rigid hardball chains with different chain lengths by the Einstein crystal method. The simulation data were in good agreement with the theoretical prediction [55]. Although the computation required by EECM in the insertion process was much more than that of the conventional Widom insertion, this method almost guaranteed successful insertions all the time and thus, reduced the insertion attempts and computation time in general.

#### 3.3.2. Rosenbluth Sampling

Long-chain molecules are extremely hard to insert in dense systems by the Widom method. A classical and feasible means was provided by Rosenbluth [26]. The long-chain molecules could be divided into multiple segments, and the whole long-chain could be inserted by growing the segments in turn. Each segment grew from the direction which made the greatest contribution to the Boltzmann factor, which was the most possible configuration of the chain to be embedded in the solution. Such biased long-chain construction process offset the segments growing orientation, which needed to be corrected by a weight factor, called Rosenbluth weight factor, every time a segment was set. Eventually, the excess chemical potential could be calculated by repeatedly constructing segments for the chain molecule and averaging all the Rosenbluth weight factors.

A large number of scholars have extended and applied this method. O’Toole and Panagiotopoulos performed Monte Carlo simulations on folding transitions of proteins on a simple cubic lattice with the Rosenbluth chain growth algorithm combined with Boltzmann weighting and multilink addition techniques [56]. Smit et al. calculated the free energy of chain molecules with this method [57]. Guo et al. carried out a parallel version of the Rosenbluth generation on GPU processors using CUDA libraries, which showed that the efficiency of this approach was almost linear with the number of CUDA cores and was limited only by hardware performance [58]. Although the calculation cost of Rosenbluth sampling in the insertion process was large, it was almost the only choice to perform successful insertions for long chains. A tricky subject was that the current Rosenbluth sampling algorithm was local and short-sighted because, for a proper chain configuration, every segment might not locate at the locally best positions. That was to say, the current chain growth algorithm would miss a significant portion of proper insertions, while the insertion was likely to fail as the chain was longer and more complex. A combination of EECM and Rosenbluth sampling was hopeful to increase the success of long-chain insertions.

### 3.4. Analysis on the Applicability of Various Biased Widom Insertion Methods for Emulsion Microencapsulation Solutions

**Figure 9:**
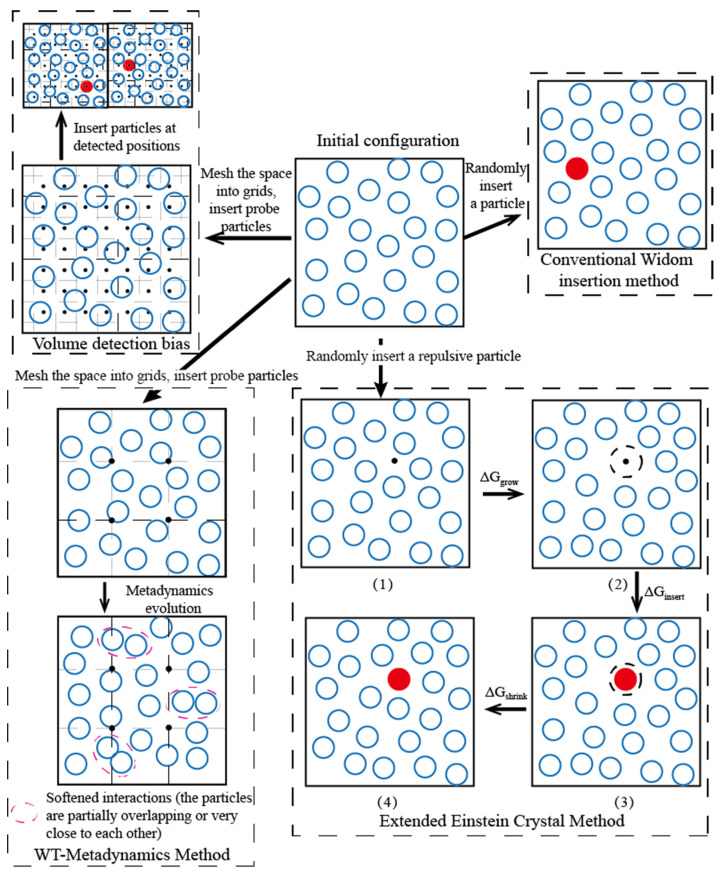
Schematic diagram of various optimized Widom methods.

A schematic diagram for various Widom-type methods is shown in Figure 9, and the applicability of these methods for dense systems is shown in Table 4. For the solutions involved in emulsion microencapsulation techniques, the chemical potentials of the O phase (polymer/alkane/aromatics) and W2 phase (surfactant/passivator/water) are particularly important. The chemical potential of a low-density system could be calculated accurately with the conventional random sampling method, which was used in the original version of the Widom method because of the high success rate of Widom insertion in the systems with sparsely distributed molecules. However, the pairwise distance of the molecules was reduced as the system density increased, and the inserted Widom test particles were often too close to other particles. Huge repulsive forces were generated, increasing the system’s potential energy to infinity. The main calculation load of the Widom insertion method lay in the multiple calculations of the internal energy change, Δ*U*, caused by the test particles. However, the insertion attempt that made Δ*U* tend to infinity was almost meaningless for the calculation of the chemical potential and caused a waste of computation resources. The three kinds of biased sampling methods described earlier all paid additional efforts in a single insertion to ensure that Δ*U* remained a finite value to the greatest extent. As a consequence, the sampling quality of spatial and ensemble averaging in Equation (2) was improved, and the total number of calculations for Δ*U* to give convergent *μ*_ex_ was effectively reduced. When choosing a biased sampling method in actual research, the increased calculation in a single Widom insertion process and the reduction of the sampling number for successful insertion configurations should be comprehensively considered. The additional calculation of the volume detection bias came from the evaluation of the detection grid points. As the system density increased, the detection grid mesh needed to be refined by the same magnitude to capture appropriate Widom insertion positions. On the other hand, the intermolecular interaction should be globally uniform to formulate unified insertion evaluation rules (such as Equation (3), based on local number density statistics). Therefore, the volume detection bias method was mainly suitable for medium- and high-density homogeneous systems and its computational advantage was gradually lost with the increase in system density. This method was suitable for the calculation of the chemical potential of small molecules in double emulsion solutions such as ion/water (W2), alcohol/water (W2), and alkane/aromatics (O).

As a newly developed ensemble bias method, the WT-Metadynamics method showed very strong system adaptability. Among all the biased optimization methods, the implementation of WT-Metadynamics was relatively difficult, in which the form of bias potential energy and the parameters involved needed to be carefully set and optimized based on the characteristics of the system. The WT-Metadynamics was proven to be suitable for dense homogeneous and heterogeneous systems, and this method was suitable for the chemical potential calculation of small molecular solvents for complex and non-uniform phases in double emulsions such as alkane/aromatics (O), polymer/aromatics (O), and polyalcohol/water (W2).

The calculation cost of EECM and Rosenbluth sampling for a single insertion was much larger than that of the Widom insertion, so these methods achieved the maximum benefit when they were applied to dense systems with macromolecules and insoluble solutes. These two methods were suitable for calculating the chemical potential of various polymers in each phase of a double emulsion, such as polymer/aromatics (O) and polyalcohol/water (W2).

## 4. Conclusions

In this paper, we focused on the complex systems in emulsion microencapsulation and reviewed suitable methods for calculating the chemical potential of solution in emulsion, and developed a new volume detection bias for dense uniform solutions. The latest progress of chemical potential calculation methods for dense solution systems was reviewed in this paper. The algorithm features, research status, and implementation results of three biased Widom methods: volume detection bias, simulation ensemble bias, and particle insertion bias were introduced and analyzed in detail. The density, uniformity, size of the molecule, and interaction complexity of the solution system were the main factors to choose a proper calculation algorithm. The main conclusions of this work were listed as follows:

(1) The main bottleneck of the original Widom method for dense systems was the low success rate to pick appropriate insertion positions with random or uniform grid sampling, which led to plenty of meaningless calculations of the system potential and, hence, reduced the calculation efficiency.

(2) The volume detection methods could be developed and customized on the basis of the original Widom method by optimizing the sampling of insertion positions with various detection methods of the configuration space. A new modification of this type of biased method was proposed and verified in this work, which was suitable for small polyatomic molecules in uniform systems, such as the aromatics solvents and alkane additives in the O-phase of microencapsulation emulsions.

(3) The WT-Metadynamics, as a recently developed simulation bias method, showed very strong system adaptability since the molecular configuration could be changed designedly, which generated appropriate positions for the Widom insertions. When a proper repulsive potential required by metadynamics was fixed, the chemical potential of complex systems could be calculated following an iteration procedure.

(4) The particle insertion bias methods were particularly suitable for the chemical potential calculation of macromolecules and insoluble molecules in dense systems. Except for the classical Rosenbluth biased chain growth method, the newly developed EECM method proposed to induce a repulsion-shrink process near the insertion position, which could also guarantee a successful insertion to improve the chemical potential calculation accuracy and efficiency in polymer solutions.

For complex solution systems, although the existing chemical potential calculation methods could meet some of the requirements of research, the chemical potential calculation of polymer systems or multicomponent systems was still very time-consuming computation work. Therefore, the development of more efficient chemical potential calculation methods is still an important topic and challenge in the field of computational physical chemistry.

## Figures and Tables

**Table 1:** Applications of the original Widom algorithm.

Researchers	System and Force Field	Molecular Configuration Sampling	Insertion Position Sampling
Wu et al. [18]	Solvent particles: ethylene oxide (200 molecules), ethanol (300 molecules) TEAM-AA force field: whole atomic force field/TraPPE-UA binding atomic force field	MC: 10^5^ molecular configurations were generated after a relaxation of 5 × 10^4^ MC steps, and one configuration sample was taken out of every 100 configurations. MD: Molecular configurations during 20 ps were generated after a relaxation of 20 ps, and one configuration sample was taken out every 20 fs	Sampling in a grid system whose grid volume was 0.5 Å^3^
Xuan et al. [27]	Ethanol (OPLS-UA) and carbon monoxide (DREIDING) at 298–323 K	MC: 5 × 10^4^ molecular configurations were generated after a relaxation of 2.5 × 10^5^ MC moves, using Towhee-7.02 software package [32]	random sampling in simulation space
Gestoso et al. [19]	Solvent: 1-magneto-4-polybutadiene chain with 30–300 monomer; Force field: COMPASS	Kinetic MC: molecular configurations during 10^−4^ s after relaxation	Sampling in a grid system whose grid volume was 0.3 Å^3^
Albo et al. [28]	64,000 carbon dioxide molecules; Force field: Isotropic Intermolecular Potential (IMP)	MD: 1000 molecular configurations during 0.75 ns were generated after a relaxation of 300 fs	random sampling in space with 2.5 × 10^6^ insertion attempts for each configuration
Coskuner and Deiters [29]	216 water molecules (SPCE, original TIP5P, and improved TIP5P) and 2 xenon atoms (LJ)	MC: 9 × 10^6^ molecular configurations were generated after a relaxation of 2.5 × 10^6^ MC moves, using HYDRO [33] software	random sampling in space

**Table 2:** Application of the volume detection bias method.

Researchers	Force Field	Molecular Configuration Sampling	Insertion Position Sampling
Khawaja et al. [36]	OPLS	MC: For each of 24 independent systems, selected 250 molecular configurations. Simulated with the Gromacs open source software [39]	Unbiased Widom sampling: 10^8^ times; Volume detection bias: 8 × 10^4^ times
Yang et al. [38]	AMBER/OPLS	MC: For each of 20 independent systems, selected 250 molecular configurations	Unbiased Widom sampling: 10^7^ times; Volume detection bias: 2.5 × 10^5^ times

**Table 3:** Results of the volume detection bias method based on the two-level criterion and detailed average.

	T	*ρ*	*μ*_ex_W_	*μ*_ex_p_	*μ*_ex_c_	*μ*_ex_	*μ*_ex_R_	(*μ*_ex_ − *μ*_ex_R_)/*μ*_ex_R_
Unit	K	kg/m^3^	kcal/mole	kcal/mole	kcal/mole	kcal/mole	kcal/mole	
H_2_O	315	991	−5.5414 ± 0.083	−0.0594	−0.3056	−5.9064 ± 0.083	−6.1244	−3.6%
	374	958	−5.2695 ± 0.127	−0.0574	−0.036	−5.3629 ± 0.127	−5.4828	−2.2%
	451	890	−4.4862 ± 0.035	−0.0523	−0.061	−4.5995 ± 0.035	−4.7748	−3.7%
Ar	320	416	−0.0222 ± 0.0005	−0.039	0	−0.0612 ± 0.0005	−0.0345	−0.027
	320	727	0.2176 ± 0.0015	−0.0681	0	0.1495 ± 0.0015	0.1582	5.5%
	320	894	0.4968 ± 0.0019	−0.0837	0	0.4131 ± 0.0019	0.3988	−3.6%

**Table 4:** Applicability of various Widom insertion methods for dense systems.

Method	Principle	Characteristics	Applicable System/Emulsion Microencapsulation Solution
Original Widom insertion method	Calculating the ensemble-averaged Boltzmann factor, 〈e^(−*β*Δ*U*)^〉*_N_* using random sampling or uniform grid sampling	For low density systems, the accuracy is acceptable. The calculation is very time-consuming, and the chemical potential accuracy is low for dense systems.	Low-density system
Volume detection bias	Inserting of probe particles to evaluate whether the detection area is suitable for particle insertions, and make intensive insertion attempts in the appropriate detected areas	The number of insertions is reduced, the accuracy of the data and the calculation efficiency is effectively improved. Inserting detection particles requires a certain amount of calculation and reasonable evaluation means need to be applied.	Uniform system of medium and high density. Ion/water (W2), alcohol/water (W2), and alkane/aromatics (O)
Simulation ensemble bias	The WT-Metadynamics applies additional external potentials to the simulation system to create appropriate insertion positions during the system evolution. Test particles are inserted at specific locations.	The algorithm skillfully constructs low-density regions for particle insertion and dynamically adjusts the system configuration according to the potential energy around the detection point. The implementation is complex.	Uniform or non-uniform complex system. Alkane/aromatics (O), polymer/aromatics (O), and polyalcohol/water (W2)
Particle insertion bias	EECM: changing the configuration near the insertion position by repulsing the nearby particles so that the test particle can be inserted successfully; Rosenbluth sampling: inserting of a long-chain molecule segment by segment, and performing of biased sampling based on the change of local internal energy in the growth direction of the chain.	The success rate of a single molecule insertion increases and the number of insertion reduces, but perform a longer time calculation for every insertion.	Dense systems with macromolecular solutes or insoluble solutes. Polymer/aromatics (O) and polyalcohol/water (W2)

## Data Availability

Not applicable.
